# Mental Distress in Patients with Cerebral Visual Injury Assessed with the German Brief Symptom Inventory

**DOI:** 10.3389/fnagi.2015.00051

**Published:** 2015-05-04

**Authors:** Carolin Gall, Doreen Brösel, Gabriele Helga Franke

**Affiliations:** ^1^Department of Rehabilitation Psychology, AHW, Magdeburg-Stendal University of Applied Sciences, Stendal, Germany; ^2^Department of Psychiatry, AMEOS Hospital, Haldensleben, Germany

**Keywords:** visual field defects, cerebral visual injury, mental health, mental distress, Brief Symptom Inventory

## Abstract

**Background:**

While there are reports on vision-related quality of life in patients with vision impairment caused by both ophthalmic and brain diseases, little is known about mental distress. In fact, mental distress after cerebral visual injury has been widely ignored.

**Methods:**

Mental health symptoms were assessed in 122 participants with visual field defects after brain damage (72 male, mean age 58.1 ± 15.6 years), who completed the German Brief Symptom Inventory (BSI) at their homes after they had been asked by phone for their participation.

**Results:**

Clinically relevant mental distress was present in 25.4% of participants with cerebral visual injury. In case of multisensory impairment, an increased amount and intensity of mental distress symptoms was observed compared to the subsample with only visual impairment.

**Conclusion:**

Assessment of comorbid mental health symptoms appears to be clinically meaningful in brain-damaged patients with visual sensory impairment. In case of clinically relevant mental distress, psychological supportive therapies are advisable especially in subjects with cerebral visual injury and comorbidities affecting other sensory modalities as well.

## Introduction

As the population ages, the number of persons affected by visual sensory impairment will increase (Rein, 2013; Boyers et al., 2015). This concerns not only visual functional loss due to eye diseases but also postretinal etiologies such as optic neuropathies of different kinds and postchiasmatic lesions mainly after stroke, tumor, and traumatic brain injury (Pogoda et al., 2012; Pollock et al., 2012; Hart et al., 2013). Frequent consequences of primary visual pathway lesions are loss of visual acuity and parts of the visual field. Thus, visual defects may lead to impairments in activities of daily living such as reading, driving, or overall orientation, i.e., task-oriented domains related to daily visual functioning (Kerkhoff, 1999). Besides task-related reduced visual functioning – which is only one domain of vision-related quality of life – the overall well-being is often reduced due to the visual impairment and especially social isolation increases in older patients (Sand et al., 2013).

In previous studies, we have shown that a higher extent of vision-related quality of life was related to lower levels of mental distress (Gall et al., 2012, 2013). Compared to mental disease, which refers to a clearly defined medical condition, the term mental distress more broadly refers to a range of mental health symptoms including depression and anxiety without implicating mental disease. In fact, path analysis revealed that these mental health symptoms are associated with task-related aspects of vision-related quality of life, i.e., subjective consequences of visual impairments on activities of daily living rather than with the size of the visual field loss which is typically determined by perimetry (Gall et al., 2013). Similarly, in a sample of low vision patients, mental distress due to vision loss was related to self-reported disability (Dreer et al., 2008). The presence of self-reported loss of visual functioning is a key aspect of diminished vision-related quality of life. Several studies have shown that reduced vision-related quality of life is a risk factor for mental distress, especially for depressive symptoms (Gall et al., 2013; Zhang et al., 2013). Thus, the maintenance of vision-related quality of life could reduce and prevent mental distress due to vision problems, and – vice versa it may be reasonably assumed that mental health interventions improve emotional-well-being in a way that also reflects in better ratings of vision-related quality of life.

Increased rates of neuropsychiatric conditions in eye diseases, especially those with a progressive development, and the danger of irreversible loss of sight such as in age-related macular degeneration (AMD) have been repeatedly reported. Mental health symptoms are not only present in patients with AMD (Brody et al., 2001; Mathew et al., 2011), but also in patients with diabetic retinopathy (Cox et al., 1998; Robertson et al., 2006; Hahm et al., 2008; Trento et al., 2013), refractive error (Owsley et al., 2007), myopia (Angi et al., 1996; Rupolo et al., 1997), and amblyopia (Koklanis et al., 2006). Such findings in eye diseases warrant further attention to vision impairment after cerebral damage, which is, however, facing the difficulty of recruiting sufficient sample sizes. So far, the presence of mental health symptoms after cerebral visual injury has been studied only in a very small sample (Gall et al., 2012; *n* = 24). Additionally, reports of multisensory impairment after brain damage are rare (Pogoda et al., 2012). Visual field loss may occur in combination with another sensory impairment, which theoretically multiplies individual difficulties complicating individual efforts to compensate for either sensory deficit. Furthermore, when the central visual pathway is injured not only sensory-specific but also heteromodal effects can be observed which may also cause altered multisensory experiences (Bolognini et al., 2013).

Since mental health symptoms in cerebral visual injury have been insufficiently investigated, we sought to fill a gap in previous research by conducting a study with the major aim to estimate the presence of mental health problems in a larger visually impaired sample using the Brief Symptom Inventory (BSI) (Franke, 2015). Furthermore, we assessed the presence of further sensory loss assuming that multisensory impairment is a risk factor for mental distress after cerebral visual injury.

## Patients and Methods

### Sample

Data of 122 subjects (72 male, mean age 58.1 ± 15.6 years) with cerebral visual injury, either due to optic neuropathies (*n* = 65) or postchiasmatic lesions after ischemic or hemorrhagic stroke (*n* = 48), took part in the study. Nine subjects were not aware of the cause of their vision impairments. Thirty-two subjects reported further sensory impairments beside visual field defects (hearing loss *n* = 19, tactile paresthesia *n* = 7, loss of sense of smell *n* = 5, *n* = 1 did not specify the additional impairment). According to the German school system, participants’ highest educational level was certificate of secondary education (*n* = 15), general/intermediate certificate of secondary education (*n* = 52), higher school certificates (*n* = 17), and academic degrees (*n* = 35). Three subjects gave no reports concerning their educational level.

The majority of study participants was married/in a partnership (*n* = 75), single (*n* = 18), divorced (*n* = 9), or widowed (*n* = 10). Ten subjects gave no information about their marital status.

Study participants were recruited out of the clinical studies data base of the Institute of Medical Psychology (Magdeburg, Germany), which is a facility for neurovisual rehabilitation. Only study participants with documented cerebral visual injury were contacted.

All subjects of the drawn convenience sample received the BSI questionnaire together with an informative letter via postal mail. Participants answered the questionnaire at their homes. All data was collected in pseudonymous form, i.e., identification codes were used to distinguish individual data records. Inclusion criteria were (i) presence of chronic visual field loss after cerebral injury, (ii) lesion age of at least 6 months, (iii) age of at least 18 years, and (iv) German as mother tongue. The study was approved by the local ethics committee and carried out in compliance with ethical standards of the Declaration of Helsinki (1964).

### Brief symptom inventory

The Brief Symptom Inventory (BSI) (Franke, 2000, 2015) is the short form of the SCL-90-R (Franke, 2014), a well-validated multidimensional symptom self-report inventory. The BSI was assessed for measuring the subjective perception of mental distress over a time period of the past 7 days. It is composed of 53 items, each of which measures distress on a 5-point Likert scale from “0 = not at all/no distress” to “4 = extremely/very strong distress.” Psychological distress is reflected in nine primary dimensions: *anger-hostility, anxiety, depression, paranoid ideation, phobic anxiety, psychoticism, somatization, interpersonal sensitivity*, and *obsessive-compulsiveness*. The number of dimensions has been confirmed by factor analyses in samples with intellectual disabilities (Kellett et al., 2004; Wieland et al., 2012). Yet, most scales were correlated and discriminant validity was shown only for the subscales *depression, anxiety* and *phobic anxiety* (Wieland et al., 2012). In fact, the scales of even shorter BSI versions with 27 items or less show satisfactory psychometric properties in comparison to the scales of the SCL-90-R (Spitzer et al., 2011; Prinz et al., 2013).

Brief Symptom Inventory raw-values between 0 and 4 were transformed into age and gender-specific normative *T*-values (normal range 50 ± 10, higher values indicating greater psychological distress) by using a standardization reference table. Additionally, three global indices of distress were calculated with *T*-scores ranging from 20 = lowest to 80 = worst psychological distress: the Global Severity Index (GSI) refers to the level of recent self-reported psychological distress and is calculated as the mean value of all item responses. The Positive Symptom Distress Index (PSDI) corresponds to the overall intensity of symptoms, which is given as an average score of all items scored above zero. The Positive Symptom Total (PST) is the number of items scored above zero, i.e., only items where a given subject reported.

By definition, cases with clinically relevant psychological symptoms were defined according to *T*-criteria. Patients were categorized as case if the *T*-values of the GSI or of at least two subscales were ≥ 63. This case definition does not indicate mental disease but the presence of subjective impairment, which is clearly above the normal range when compared to healthy subjects and thus considered clinically relevant.

### Statistical analysis

Demographic and clinical data were analyzed with SPSS 21.0 for Windows (IBM Deutschland GmbH, Ehningen). Data for men and women were analyzed together since there were no significant differences regarding sociodemographic as well as BSI data. Data were tested for normal distribution with the Kolmogorov–Smirnov test. All scales of the BSI were skewed to the right. Between subjects comparisons were conducted with the Mann–Whitney *U*-Test. In order to avoid alpha error accumulation, Bonferroni-correction was conducted and *p*-values of 0.05/12, i.e., below 0.042 were considered as statistically significant. All tests were two-tailed. Correlations (Kendall’s tau-b) between global indices of the BSI and demographic variables were calculated.

## Results

Mean raw and *T*-scores of BSI subscales are shown in Table 1. *T*-values ranged between 49.43 (anger-hostility) and 53.26 (phobic anxiety). A total of 31 (25.4% of the sample) subjects presented with clinically relevant psychological symptoms assessed with the BSI according to *T*-criteria. Concerning only the GSI, 12 subjects (9.8% of the sample) presented with *T*-values equal or above 63. BSI global indices did not correlate significantly with demographic variables (all correlations with sex below 0.2, all correlations with age were below 0.1).

**Table 1:** **BSI results and number of cases with mental distress exceeding the normal *T*-range**.

BSI scale/index	Raw score	*T*-value	*T*-value > 60		Range of *T*-values
	*M* (±SD)	*M* (±SD)	*n*	(%)	
Anger-hostility	0.30 (±0.47)	49.43 (±9.82)	16	13.11	35–80
Anxiety	0.32 (±0.44)	50.36 (±9.29)	22	18.03	37–75
Depression	0.30 (±0.45)	50.11 (±8.43)	18	14.75	38–74
Paranoid ideation	0.37 (±0.49)	49.90 (±8.49)	15	12.30	39–74
Phobic anxiety	0.27 (±0.45)	53.26 (±9.57)	33	27.05	43–80
Psychoticism	0.22 (±0.39)	50.75 (±7.96)	15	12.30	41–74
Somatization	0.41 (±0.41)	51.54 (±9.45)	20	16.39	37–74
Interpersonal sensitivity	0.38 (±0.55)	50.35 (±9.32)	19	15.57	39–80
Obsessive-compulsiveness	0.54 (±0.48)	50.83 (±9.26)	24	19.67	33–69
Global Severity Index	0.35 (±0.35)	50.20 (±10.06)	23	18.85	28–73
Total number of positive symptoms	13.97 (±11.06)	50.52 (±10.31)	25	20.49	31–74
Positive Symptom Distress Index	1.20 (±0.40)	50.41 (±9.42)	18	14.75	28–80

**T* > 60 is an indicator for mental distress that deviates from the standard, whereas *T* > 63 is used in order to identify subjects according to BSI case definition*.

The comparison of mental distress between subjects with multisensory impairment vs. subjects with visual field defects only is shown in Table 2. In the group with multisensory impairment, a higher intensity of mental distress was observed (PSDI: *Z* = 826.5, *p* < 0.01). Additionally, the total number of positive symptoms, PST, was significantly larger in the group with multisensory impairment amounting at 18.71 (±11.93) compared to subjects with only visual impairment 12.35 (±10.33) (*Z* = 927.5, *p* < 0.01). Further, the GSI significantly differed between groups (*Z* = 890.5, *p* < 0.01). Subscale differences between subjects with multisensory vs. only visual impairment were observed for all subscales except *paranoid ideation, phobic anxiety*, and *Psychoticism* (Table 2).

**Table 2:** **Comparison of BSI *T*-values between subjects with multisensory impairment and only visual impairment**.

BSI scale/index	Median of subjects with multisensory impairment	Median of subjects with visual impairment only	Mann–Whitney *U*	(*p*)
	(*n* = 31)	(*n* = 91)	
Anger-hostility	73.76	57.32	1030.5	0.024
Anxiety	76.34	56.45	950.5	0.007
Depression	77.50	56.05	914.5	0.003*
Paranoid ideation	67.08	59.60	1237.5	0.302
Phobic anxiety	72.08	57.90	1082.5	0.050
Psychoticism	68.00	59.29	1209.0	0.227
Somatization	75.89	56.60	964.5	0.009
Interpersonal sensitivity	74.45	57.09	1009.0	0.017
Obsessive-compulsiveness	73.65	57.36	1034.0	0.027
Global Severity Index	78.27	55.79	890.5	0.002*
Total number of positive symptoms	77.08	56.19	927.5	0.004*
Positive Symptom Distress Index	80.34	55.08	826.5	0.001*

*Significant difference after Bonferroni correction.

## Discussion

Since the population ages, along with an increased occurrence of visual sensory impairment after cerebral visual injury, the adverse effects of comorbid mental health symptoms are likely to increase. The present study is the first preliminary investigation of the presence of mental distress in a larger sample of subjects who have suffered cerebral visual injury, either due to optic nerve lesions or posterior parts of the visual pathway resulting in chronic visual field defects.

Clinically relevant mental distress in this visually impaired patient group was present in about 25% of participants. The majority of the sample reported no clinically relevant impairment in BSI subscales resulting in a right-skewed distribution; however, it remains possible that the occurrence mental distress was underestimated since all study participants met the inclusion criteria for neurovisual rehabilitation and did not present with acute psychiatric conditions. Furthermore, the sample does not contain visually impaired patients living in nursing homes or who are legally blind because of extensive visual field defects. To some extent this may have caused biased results in a way that mental distress was underestimated when compared to the population of visually impaired patients after cerebral injury.

Late-life mental disorders are likely to increase (Westphal et al., 2010) and age-related sensory impairment appears to constitute one of the risk factors (Gall et al., 2013). Kempen et al. (2012) reported a higher level of depression and anxiety as well as lower health-related quality of life in elderly subjects with vision impairments. In the present study, however, we could not observe an association between age and self-reported mental distress. This may be due to the fact that the sample consisted of patients with largely heterogeneous cerebral visual injury concerning the extent of the visual field loss. While the occurrence of any cerebral visual injury is more likely with increasing age, the extent of visual field loss itself is not correlated with age. Thus, in future studies, the extent of the visual field loss should be considered as a covariate.

In the present study, we have shown that an additional sensory impairment (most subjects reported hearing problems beside vision problems) goes along with increased psychological distress in all BSI components except *paranoid ideation, phobic anxiety*, and *Psychoticism*.

This finding is in line with the literature on dual sensory loss, i.e., combined hearing and vision loss, which has been recently reviewed by Heine and Browning (2014). Based on only seven available studies, they concluded that dual sensory loss is related to an increased prevalence of depression while data on the prevalence of anxiety was not available. In fact, the present study provides evidence that besides *Depression* also, other BSI components were elevated terms of increased psychological distress, namely, *anger-hostility, anxiety, somatization, interpersonal sensitivity*, and *obsessive-compulsiveness*. Thus, a deeper investigation of clinical manifestations not only of anxiety appears to be necessary in future large-scale studies. Additionally, personality changes after cerebral visual injury have been already reported (Jobke et al., 2008). For future studies, the combined use of the BSI or the long-version SCL-90 together with a personality inventory should be considered and diagnoses of mental disorders should be made available, which requires a more extensive study.

Tools such as the BSI allow for a multidimensional assessment of mental distress and thus, represent a way to screen for and assess emotional distress including anxiety and depression. In any case, when clinically relevant mental distress is present, e.g., according to BSI results, this finding may encourage the clinician to obtain an accurate clinical diagnosis to finally provide supportive psychotherapeutic interventions. Treatment of mental health symptoms in patients with visual and multisensory impairment requires the collaboration of a number of professional disciplines. However, reports on rehabilitation options are limited. Techniques such as telepsychiatry, internet-delivered therapy programs, and bright-light therapy appear to be feasible for subjects with mental health problems due to visual impairment since these were already used in managing symptoms associated with depression (Westphal et al., 2010). Cognitive behavior therapy adapted for patients with acquired brain injury has already shown positive effects concerning mood and community integration (Bradbury et al., 2008; Arundine et al., 2012). Future efforts should focus on identifying vision – respectively multisensory impaired patients with mental health symptoms and provide targeted psychological treatment in an ambulatory therapeutic setting as well. Within this context, diagnostic instruments such as the BSI can be used to support determining psychiatric caseness. Vision-related quality of life questionnaires such as the National Eye Institute Visual Functioning Questionnaire (NEI-VFQ) (Mangione et al., 1998, 2001) enable physicians and therapists to obtain a detailed profile of self-reported visual functioning and socioemotional consequences, which may provide helpful information and impulses for psychosocial interventions. The NEI-VFQ can be assessed in subjects with cerebral visual injury as well, though it has been originally constructed to assess vision-related quality of life in ophthalmic subjects (Raphael et al., 2006; Papageorgiou et al., 2007; Gall et al., 2009, 2010).

Recently, Rovner et al. (2014) have conducted the first randomized trial comparing whether either behavior activation, i.e., a training of adaptive behaviors, or supportive therapy, i.e., a non-directive psychological treatment that provides emotional support, combined with low vision rehabilitation better prevents depressive disorders in patients with AMD. In their study, the NEI-VFQ was used together with the Patient Health Questionnaire-9 to assess depressive symptoms. When compared to standard outpatient, low vision rehabilitation the incidence of depressive symptoms was clearly reduced after both mental health interventions. After low vision rehabilitation combined with behavior activation, depressive symptoms were prevented in a way that social participation was maintained. However, also functional vision improved to a larger extent in this group and between group differences were not observed. These encouraging reports document that besides low vision training (e.g., Pouget et al., 2012; Amore et al., 2013) or neuropsychological rehabilitation approaches such as vision restoration training (Kasten et al., 1998; Gall et al., 2008), psychological supportive therapies are advisable especially for subjects with cerebral visual injury and comorbidities affecting other sensory modalities as well.

## Conflict of Interest Statement

The authors declare that the research was conducted in the absence of any commercial or financial relationships that could be construed as a potential conflict of interest.
